# Geometric Implications of Photodiode Arrays on Received Power Distribution in Mobile Underwater Optical Wireless Communication

**DOI:** 10.3390/s24113490

**Published:** 2024-05-28

**Authors:** Tharuka Govinda Waduge, Boon-Chong Seet, Kay Vopel

**Affiliations:** 1 Department of Electrical and Electronic Engineering, Auckland University of Technology, Auckland 1010, New Zealand; boon-chong.seet@aut.ac.nz; 2 School of Science, Auckland University of Technology, Auckland 1010, New Zealand; kay.vopel@aut.ac.nz

**Keywords:** photodiode array, received power distribution, mobile optical links, underwater optical wireless communication, underwater vehicles

## Abstract

Underwater optical wireless communication (UOWC) has gained interest in recent years with the introduction of autonomous and remotely operated mobile systems in blue economic ventures such as offshore food production and energy generation. Here, we devised a model for estimating the received power distribution of diffused line-of-sight mobile optical links, accommodating irregular intensity distributions beyond the beam-spread angle of the emitter. We then used this model to conduct a spatial analysis investigating the parametric influence of the placement, orientation, and angular spread of photodiodes in array-based receivers on the mobile UOWC links in different Jerlov seawater types. It revealed that flat arrays were best for links where strict alignment could be maintained, whereas curved arrays performed better spatially but were not always optimal. Furthermore, utilizing two or more spectrally distinct wavelengths and more bandwidth-efficient modulation may be preferred for received-signal intensity-based localization and improving link range in clearer oceans, respectively. Considering the geometric implications of the array of receiver photodiodes for mobile UOWCs, we recommend the use of dynamically shape-shifting array geometries.

## 1. Introduction

Interest in autonomous and connected mobile systems for underwater applications is growing. With the rising global human population and demand for food, energy, and other resources, the utilization of oceans for economic activities, also referred to as the blue economy, has garnered the attention of researchers and governments globally. Open-water aquaculture, offshore energy farms, deep-sea mining, and coastal tourism are some examples. The U.S. Department of Energy has forecasted growth of USD 3–8 bn from 2020 to 2030 in the local underwater vehicle docking and recharging market to allow unhindered year-round underwater observations without the need to surface for data collection or recharging [1].

It is preferred to use untethered data transmission with unmanned underwater vehicles (UUVs) to reduce costs and entanglement, and avoid otherwise possible hydrodynamic forces on cables that affect maneuverability [2]. While acoustic communication methods are mature and capable of covering vast distances, they offer low channel bandwidth and data rate. On the other hand, radio frequency signals can be severely attenuated due to the high conductivity of seawater and large antennas needed for lower-frequency and higher-power transmissions are physically cumbersome for compact UUVs with limited energy budgets. This leaves underwater optical wireless communication (UOWC) as a suitable candidate from a size-, bandwidth-, data-rate-, and efficiency-based perspective [3]. However, optical links are prone to link loss due to misalignments due to line-of-sight (LOS) requirements. Further, their signal attenuation coefficient is higher than that of acoustic links and a function-of-light wavelength, implying that a robust mobile optical link may need continuous determination of channel state even for slow-moving UUVs.

Although the effects of seawater on the attenuation of the optical signal cannot be easily circumvented, a large field of view (FOV) or large receiver aperture could aid in reducing geometrical losses. This could be supplemented by enhancing the gain through wireless communication diversity strategies and dynamic intelligent alignment between receiver and transmitter. However, the independent movements of both receiver and transmitter introduce another layer of complexity to the problem. Addressing these challenges requires solutions that account for the unique characteristics of transmitters and receivers and the constraints of the underwater environment.

The goal of this contribution is threefold:

- To provide a thorough review of the existing literature and commercial products of UOWC transmitter and receiver designs, with a focus on mobile UOWCs.
- To devise a model for estimating the received power distribution of diffused line-of-sight mobile optical links that accurately reflects the intensity distribution profiles of LEDs with non-uniform or irregular polar-intensity distributions.
- To conduct a spatial analysis using the above model to investigate the parametric influence of the placement, orientation, and angular spread of PD positioning on array-based receivers for mobile UOWC in different Jerlov seawater types. We assess this for wavelengths within the visible spectrum (400–700 nm), and we compare the results for the overall received power and bit-error rates, employing modulation schemes commonly used for diffused line-of sight optical links. The results highlight the significance of designing dynamically shape-changing receiver arrays.

The rest of the paper is organized as follows. In Section 2, we review the related works to date on transmitter and receiver designs aimed at mitigating misalignments from a physical design perspective. Specifications of existing commercial devices have also been presented here. In Section 3, we introduce the preliminary concepts and channel models used for UOWCs. In Section 4, we delineate our channel model, and then, in Section 5, we use this model to outline the parameters, limitations, and design of the geometric analysis. Section 6 presents a discussion of the insights observed with supporting results. Finally, Section 7 concludes the article with a summary of our key findings.

## 2. Literature Review

In wireless communication, achieving a near-perfect transmission of energy from transmitter to receiver is challenging. This is particularly difficult in UOWC, due to inherent and environmental factors such as signal attenuation, misalignment, obstruction, reflection, refraction, and ambient noise. To overcome these challenges, strategies are needed to both transmit and capture photons effectively. While researchers often focus on strategies such as increasing the transmitting power of the signal and the area of photodetection, or addressing signal losses from attenuation and refraction by using complicated signaling strategies [4], state-of-the-art hardware-based strategies are underexplored.

### 2.1. Transmitters

**Figure 1:**
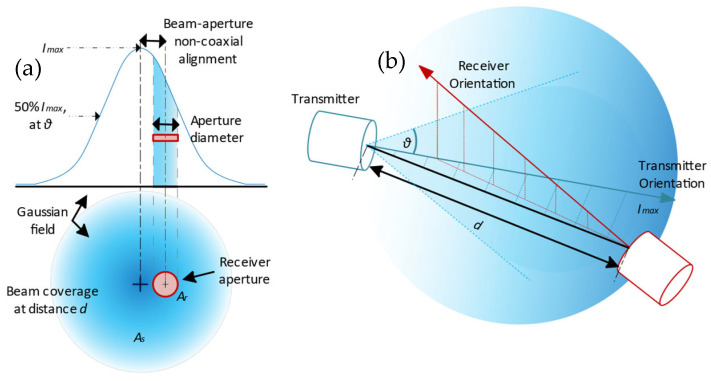
(**a**) The polar distribution of the emitter’s optical intensity is non-uniform, especially for diffused line-of-sight optical links; the received power is contingent on multiple factors. (**b**) a miniaturized visual of a typical transmitter–receiver alignment expected for a mobile UOWC. Here, θ is the beam divergence angle and d is the separation between the transmitter and the receiver.

Light-emitting diodes (LEDs) and laser diodes are the main candidates for transmitters considering their ability for light modulation. However, a mobile UOWC requires a significant region of coverage, where LED-based emitters with large beam-divergence angles are preferred. Therefore, unfortunately, these links have a much lower modulation bandwidth of up to several tens of MHz, limited by the cut-off ceiling of the LEDs and driver circuitry used, despite the fact that the visible light spectrum spans several THz in frequencies [4]. Such connections are called diffused line-of-sight (DLOS) links. The resultant light field’s intensity distribution is often non-uniform but may take on a Gaussian, Lambertian, or even an irregular (e.g., batwing) distribution around the emitter’s center axis (axis of orientation). In such cases, the angle between the receiver and the emitter’s axis becomes an additional variable (Figure 1). Considering a realistic DLOS link, the beamwidth in Figure 1 at the distance of the receiver will be much larger than the receiver diameter, and the variation of optical power received along the plane of the photodetector aperture more even.

To mitigate this non-uniformity, Han et al. devised a transmitter employing a blue LED array with a free-form lens to achieve a 150° angle of divergence with 90% uniform intensity, demonstrating a data rate of 19 Mbps over an 8 m distance with an attenuation coefficient of 0.4 m^−1^ [5]. Similarly, Tong et al. developed a quasi-omnidirectional triangular prismatic array of three LED modules, each with nine LEDs, achieving a full 360° intensity distribution with a peak-value illuminance of only 1 dB higher than the trough value. They achieved a data rate of 22 Mbps over a 10 m distance, suggesting applicability for swarm robotics or an underwater optical cellular network [6]. Liu et al. investigated the placement of 16 LEDs, comparing a rectangular layout, a particle swarm optimized (PSO) layout, and a hybrid layout for achieving uniform power distribution across the depths of clear and turbid waters. Their results demonstrated a significant reduction in power distribution variance with the PSO layout, indicating consistent emission uniformity across the water classifications [7]. In addition to this, to maintain consistent alignment, Romdhane and Kaddoum proposed a novel beam adaptation algorithm for UOWC receivers based on artificial intelligence reinforcement learning to optimize beamwidth and orientation in different water types for point-to-point line-of-sight (P2P-LOS) communication [8]. They compared the performance of Q-learning and State-Action-Reward-State-Action (SARSA) algorithms, with SARSA converging more quickly. Additionally, Cui et al. presented work on pointing adjustable beam arrays using reflectors and lasers to enhance performance under varied water conditions, resulting in reduced path loss and improved adaptability to attenuation and turbulence [9]. As an alternative approach, Yildiz et al. have explored the deployment of underwater reflectors to improve the gain by the inclusion of non-line-of-sight (NLOS) paths, yielding promising outcomes [10]. They indicate that using mirrors between the transmitter and receiver could potentially yield a maximum gain of approximately 3 dB in clear water, and further propose that utilizing the air/water boundary as a reflector might offer greater efficiency compared to traditional mirrors due to higher reflectance. However, the overall benefit of deploying such reflectors in mobile scenarios may be low, as they could contribute to environmental clutter and unwanted reflections. In such cases, using a tether may be more practical. Nevertheless, integrating NLOS paths could prove advantageous for UOWC applications, particularly in scenarios where UUVs need to traverse environments where LOS connections are unattainable, such as in trenches or caves.

### 2.2. Receivers

However, research works in addressing those obstacles from the receiver’s perspective appear to be comparably lacking. This is particularly critical for mobile receivers, where adjustments at the receiver could theoretically reduce misalignments, improve gain, and allow the device a greater degree of autonomy. At the material level, halide perovskite (HP) polymer-based photodetectors have garnered increasing attention in recent years due to their cost-effective manufacturing and favorable semiconductor properties [11]. These detectors offer expansive FOV and are readily scalable, overcoming the trade-offs between modulation bandwidths, detection regions, and viewing angles [12]. Yu et al. demonstrated the broadband photo-response of a custom-designed HP, indicating enhanced stability of the photodetector in water, along with superior optical performance, achieving a peak external quantum efficiency of 630% at 644 nm and a remarkably high responsivity of 3.27 A/W [11]. Similar performance results are exhibited by Zhou et al., where a photo-response of 3.93 A/W for 520 nm, and an extremely low noise level of $1.26\times 10^{-12}$ A/√Hz, was observed, although at a lower baud rate of 128 kbps [13]. Moreover, Kang et al. demonstrated that HP-based polymer fibers could serve as omni-directional and flexible “photon-collectors”, with an Avalanche Photodiode (APD) serving as the receiver. Employing a non-return-to-zero on-off keying (NRZ-OOK) and quadrature amplitude modulation–orthogonal frequency division multiplexing (QAM-OFDM) techniques, data rates of 23 Mbps and 152.5 Mbps were achieved across a 1 m long tank, respectively [12]. Xu et al. have further demonstrated that HPs could be enhanced with self-healing properties underwater, showing that they may be very beneficial for long-term underwater deployments [14]. Performance results are provided for UOWCs while in damaged conditions and post-self-healing [14]. Nonetheless, the use of HPs for photodetection is a novel research field and it has yet to mature for integration into prototypes for field tests in mobile UOWCs.

As alternative high-gain devices with large FOVs and reduced alignment requirements, photomultiplier tubes (PMTs) offer promise. However, their susceptibility to ambient light and vibrations renders them impractical for mobile underwater solutions without additional interventions [15]. Alternatively, semiconductor-based photodetectors such as PIN photodiodes (PIN-PDs) or APDs are preferred. They feature planar detection regions sensitive to the angle of incidence (AOI) of photons. Therefore, in dynamic mobile communications scenarios where transmitter–receiver configurations are non-coaxial, design decisions are crucial to maintain an appropriate AOI within the detector’s FOV. Many existing studies have explored the use of such photodetector arrays, typically in fixed geometric orientations that are either flat or curved/spherical. Lin et al. investigated the feasibility of employing a green-centered InGaN micro-LED array as both transmitter and receiver, achieving a modulation bandwidth of 251.3 MHz and a data rate of 660 Mbps in a full-duplex link at 450 nm spanning 2.3 m [16]. They also demonstrated increased photodetector sensitivities with negative biasing and proposed utilizing this array as a photovoltaic cell in a charging system which may help prolong underwater deployments. However, despite the many advantages, they demonstrate a quantum efficiency of 8.20% (−5 V bias and responsivity of 0.0259 A/W) for a light power density of 56.8 W/m^2^, which seems very inefficient if immediately adopted for mobile UOWCs. In another study, Liu et al. designed a receiver featuring a Fresnel lens array to enhance coverage, achieving a maximum modulation bandwidth of 50 MHz and a data rate of 100 Mbps with a pre-equalization circuit specifically tailored for mobile UOWCs [17]. Despite this, detailed performance metrics regarding receiver orientation have not been provided. Moreover, the use of Fresnel lenses may result in the loss of initial directional information due to light convergence. Simpson et al. developed a quasi-omnidirectional smart transceiver utilizing a 3D spherical arrangement of lenses to focus light onto a planar array of photodiodes [18]. This transceiver exhibits a quasi-omnidirectional FOV and the ability to estimate angle-of-arrival using a pattern-matching algorithm accurate to ±20° at the best resolution, demonstrating electronically switched beam steering. They use a static design for the receiver array; however, it is mounted on servo motors in a “pan-and-rotate” system. Likewise in 2012, Rust and Asada tested a fixed, circular photodiode array for integrated communication and localization [19]. They controlled a remotely operated vehicle using remotely transmitted thrust commands. In addition, Table 1 further summarizes a survey of the existing commercial transceivers by their port shapes and performance (information on the receiver type used, PMT, is only publicly available for Sonardyne devices).

Despite extensive research on transmitters in underwater optical wireless communication (UOWC), there appears to be a lack of coherent and goal-oriented research regarding receivers. From an engineering perspective, an ideal mobile UOWC receiver should fulfill three main tasks: ensuring spatially reliable communication, achieving strong link quality, and enabling localization [20]. This discrepancy prompts questions about whether the lack of coherence is due to a lack of preliminary analyses conducted on the performance of photodetector array-based receivers in UOWC. To our knowledge, analyses that factor in the numerous determining parameters which include water quality, geometric losses, transmission factors, and photodetector characteristics have not been conducted. We conduct our analysis in hopes of bridging this gap and providing insights towards more goal-oriented UOWC arrayed-receiver designs.

**Table 1:** Specifications of commercially available UOWC receivers and transmitters.

Manufacturer	Model	Transmitter Type	Color	Range (m)	Data Rate (max)	Receiver Port Shape	Power Consumption (W)
Aquamodem [21]	OP2/OP2L	LED	Cyan	1	80 kbps	Flat	Not available
Sonardyne [22]	8361 Bluecomm 200	LED	Blue	150	10 Mbps	Dome	Receiver: 10 W Transmitter: 15 W
	8361 Bluecomm 200 UV	LED	UV	75	10 Mbps	Dome	Receiver: 10 W Transmitter: 30 W
Hydromea [23]	Luma 100	LED	Blue	2	115 kbps	Flat, potted	Receiver: 0.5 W Transmitter: 1–2 W
	Luma 250LP	LED	Blue	7	250 kbps	Flat, potted	Receiver: 0.5 W Transmitter: 2–5 W
	Luma 500ER	LED	Blue	50	500 kbps	Flat, potted	Receiver: 1 W Transmitter: 2–5 W
	Luma X	LED	Blue	50	10 Mbps	Flat	Receiver: 2 W Transmitter: 2–17 W
	Luma X UV	UV LED	UV	50	10 Mbps	Flat	Receiver: 2 W Transmitter: 2–17 W

## 3. Preliminaries

**Figure 2:**
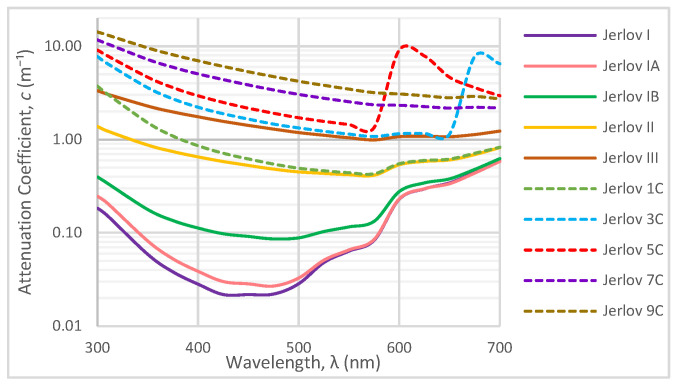
Spectral attenuation variation of Jerlov water types [25].

Underwater optical signals experience a wavelength (λ)-dependent attenuation $c\left(\lambda \right)$, which is a linear sum of absorption $a\left(\lambda \right)$ and scattering $b\left(\lambda \right)$ coefficients as shown in Equation (1) based on the composition of water. The magnitudes of light scattering and absorption may be used to categorize water into Jerlov types I, IA, IB, II, and III, which are oceanic waters, and types 1C, 3C, 5C, and 7C, which are coastal waters of increasing turbidity. Their spectral distributions for attenuation values are given in Figure 2. One limitation of the Jerlov classification is the assumption that the water body is homogeneous, which may not hold in a realistic scenario of underwater optical communication if the optical link crosses the physical boundaries (pycno-, halo-, and thermoclines) that structure coastal and open-ocean systems [24]. The nominal absorption, scattering, and attenuation coefficient values for clear, coastal, and turbid waters are given in Table 2.

The attenuation of the initial light intensity $I_{0}$ over a distance d follows the Beer–Lambert law as shown in Equation (2). The power transmitted $P_{t}$ is evaluated as Equation (3), where $P_{e}$ is the electrical power and $\eta_{e}$ is the optical power conversion efficiency. The power $P_{r}$ received by a photodiode of surface area $A_{r}$, and inclination angle β, is given by Equation (4), where $A_{s}$ is the geometric surface over which it is distributed and $I_{0}=\frac{P_{t}}{A_{s}}$. $A_{s}$ is commonly estimated as Equation (5) for P2P-LOS and DLOS links where the emitter beam divergence angle θ (the angle at which intensity is 50% of its maximum) is much smaller than $\frac{\pi }{20}$ or is greater than $\frac{\pi }{20}$, respectively [26]. Substituting (5) in (4) derives a model for $P_{r}$ as Equation (6).

(1) $$c\left(\lambda \right)=a\left(\lambda \right)+b\left(\lambda \right)$$

(2) $$I_{d}=I_{0}e^{-c\left(\lambda \right)}$$

(3) $$P_{t}=\eta_{e}P_{e}$$

(4) $$P_{r}=\left(\frac{P_{t}}{A_{s}}\right)e^{-c\left(\lambda \right)d}A_{r}\cos\left(\beta \right)$$

(5) $$A_{s}=\left\{\begin{array}{c} \pi {\left(d\tan\theta \right)}^{2}\;\;\;\;\;\;\;\;\;\;\;\;\;\;\left(\theta \ll \frac{\pi }{20}\right)\\ 2\pi d^{2}\left(1-\cos\theta \right)\;\;\;\;\left(\theta >\frac{\pi }{20}\right) \end{array}\right.$$

(6) $$P_{r}=\left\{\begin{array}{c} \left(\frac{\eta_{e}P_{e}}{\pi d^{2}\tan^{2}\theta }\right)e^{-c\left(\lambda \right)d}A_{r}\cos\beta \;\;\;\;\;\;\;\;\;\;\;\;\;\;\;\;\;\;\;\left(\theta \ll \frac{\pi }{20}\right)\\ \left(\frac{\eta_{e}P_{e}}{2{\pi d}^{2}\left(1-\cos\theta \right)}\right)e^{-c\left(\lambda \right)d}A_{r}\cos\beta \;\;\;\;\;\;\;\;\;\;\;\left(\theta >\frac{\pi }{20}\right) \end{array}\right.$$

## 4. Devised Model for Received Power

The geometric surface $A_{s}$ for a DLOS link in the literature is commonly identified with that of a sphere [20] or a spherical cap [26], where the former assumes that the intensity distribution is uniform, and the latter tends to overestimate the received power by assuming a uniform distribution within the beam divergence angle but negating any power dissipation beyond the half angle θ. It is also common to generalize LED emission to Lambertian radiation patterns [27]. However, unlike in indoor free-space optical wireless communication systems where the fixed nodes are on the roof, and the mobile nodes often move on a parallel plane below (the floor), mobile UOWC consists of the UUVs moving in the third axis as well. Since LED-based emitters are preferred for DLOS links, idealized approximations like those mentioned above may not be accurate for determining the received power within the established link range. This is because even a slight change in the polar angle at the transmitter can result in a significant region projected at the receiver end. Moreover, manufacturers typically only supply information regarding the overall transmitting optical power and the polar distribution curve of the relative intensity normalized to the LED’s center axis. This makes it difficult to determine and adopt the actual polar intensities of non-uniform or irregular patterns into the DLOS UOWC channel models. In addition to this, β in Equation (4) is true only when the reference view of the axes of the emitter and receiver are coaxial. Therefore, herein we have adapted Equation (4) to approximate the received power more accurately in a mobile link scenario. For simplicity, we assume that the efficiency of emission is a uniform property across all angles at 100%, i.e., $\eta_{e}=1$.

**Figure 3:**
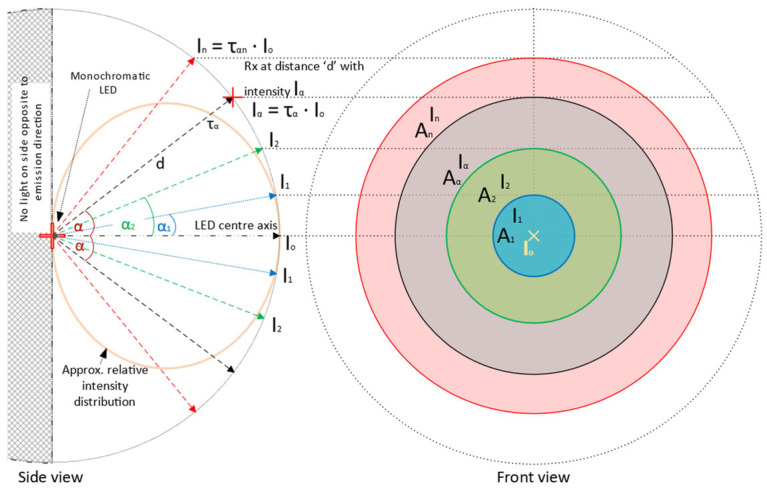
Relative intensity distribution over annuli concentric about the LED center axis.

The hemispheric area illuminated by the LED as the light source is divided into N annuli concentric about the LED’s center axis as shown in Figure 3. The intensity $I_{n}$ of the $n_{th}$ annulus (n=1…N) is determined at distance d from the point of emission that subtends an angle $\alpha_{n}$ with the center axis and has a surface area $A_{n}$, where τ is the corresponding relative intensity extracted from the datasheet. The total transmit power $P_{T}$ is given by the sum of powers at each of the N annuli, corresponding to the number of samples from the intensity distribution. With receiver (Rx) and transmitter (Tx) being non-coaxial, Equations (7)–(13) illustrate the derivation of the adapted total transmit power, while Equation (14) gives the power received by the receiver PD at each annulus:

(7) $$I_{n}=\tau_{\alpha_{n}}I_{0}$$

(8) $$A_{\alpha_{1}}=2\pi d^{2}\left(1-\cos\alpha_{1}\right)$$

(9) $$A_{\alpha_{2}}=2\pi d^{2}\left(1-\cos\alpha_{2}\right)$$

(10) $$A_{2}=A_{\alpha_{2}}-A_{\alpha_{1}}\to A_{n+1}=A_{\alpha_{n+1}}-A_{\alpha_{n}}$$

(11) $$A_{n}=2\pi d^{2}\left(\cos\alpha_{n-1}-\cos\alpha_{n}\right)\;;\;\left(\alpha_{0}=0^{{}^{\circ}}\right)$$

(12) $$P_{n}=I_{n}A_{n}=\tau_{\alpha_{n}}I_{0}A_{n}$$

(13) $$P_{T}=\sum_{n=1}^{N}P_{n}={2\pi d^{2}I}_{0}\sum_{n=1}^{N}\tau_{\alpha_{n}}\left(\cos\alpha_{n-1}-\cos\alpha_{n}\right)$$

(14) $$P_{r_{\alpha }}=\tau_{\alpha }I_{0}e^{-c\left(\lambda \right)d}A_{r}\cos\beta$$

where α is the angle subtended at Tx by the ray and center axis of Tx, and β is the inclination of the Rx to the horizontal axis parallel to and facing the Tx including any rotation; their linear sum gives the overall AOI, φ, as shown in Equation (15). Rearranging Equation (13) to express $I_{0}$ in terms of other quantities and substituting it into Equation (14), we obtain Equation (16) for the adapted received power at α. This model considers the symmetry of the intensity distribution about the LED’s center axis. Thus, it simplifies a 3D distribution to just the plane-angle measure α, where an Rx at any cartesian coordinate within the annulus of interest and whose arctan argument to the center axis of Tx is approximately α will demonstrate the same received power when revolved around the center axis. Using this model, we will study the geometrical implications for optimizing the received power for a PD array in the next section.

(15) φ=α+β

(16) $$P_{r_{\alpha }}=\left(\frac{{\tau_{\alpha }P}_{T}}{2\pi d^{2}\sum_{n=1}^{N}\tau_{\alpha_{n}}\left(\cos\alpha_{n-1}-\cos\alpha_{n}\right)}\right)e^{-c\left(\lambda \right)d}A_{r}\cos\varphi$$

## 5. Geometric Implications for Optimizing PD Arrays for Received Power

Equation (16) illustrates the dependency of received power on AOI *φ*, attenuation coefficient c, and relative light intensity at location of receiver $\tau_{\alpha }$. However, the level of influence of each feature in mobile UOWC may be more dynamic based on the environmental properties. While PD arrays have often been used to improve the signal-to-noise ratio (SNR), the geometric implications for PD placement are unclear. Here, we simulate the effect of PD placement under different directional, rotational, and attenuation parameters as applicable to mobile UOWC.

### 5.1. Parameters and Simulation Design

**Figure 4:**
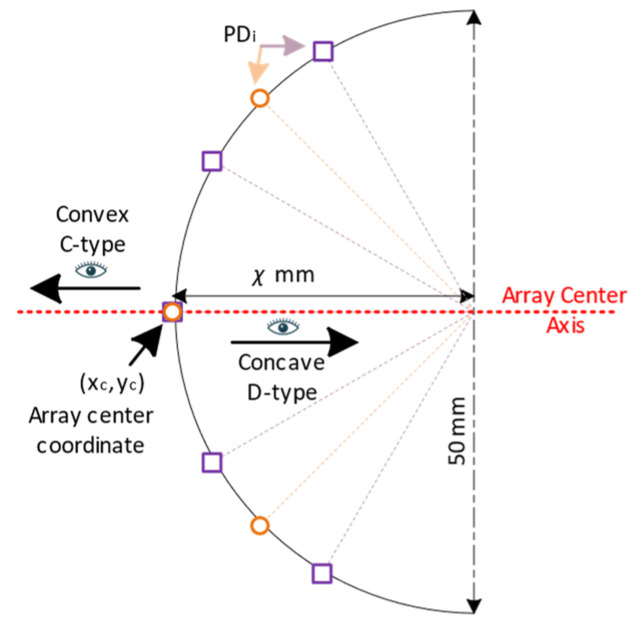
Array configuration and naming convention. Array center axis is orthogonal to the tangent to curve at the middle PD, with coordinate $\left(x_{c},\;y_{c}\right)$. C-type and D-type arrays observe the light from the outside and inside of the curve, respectively. Orange circles and purple squares are representative of the PD placements of the 3PD and 5PD arrays, respectively. Each PD is equidistantly placed along the curve starting from the array center.

Two arrays, one with three and the other with five PDs, are evaluated in both convex (C-type) and concave (D-type) configurations (Figure 4). In both configurations, the inclination of PD placement along an ellipse is varied by the distance of the vertex. The degree of convexity or concavity increases from a flat (F-type) array to depths of 10, 25, 40, and 55 mm. These arrays are labeled as Cχ or Dχ, where χ refers to the depth of convexity or concavity. For example, C25 refers to a convex array with a depth of 25 mm. The PDs are spaced equidistant along the curve. The co-vertex is 50 mm in each case. The concave and convex arrays have the PDs placed inside and outside of the transparent curved surface, respectively. The LED intensity distribution pattern and PD parameters used are from real-world devices: an Osram LZ4-00G108 LED [28] and an OSI Optoelectronics SD200-12-22-041 Silicon PD [29], respectively.

For each PD, the reference noise-equivalent power (NEP) is $1.6\times 10^{-13}$ W/√Hz [29]. The cumulative typical noise power $P_{\eta_{tot}}$ at the modulation frequency (BW) can be estimated as Equation (17), where $I_{PD}$ is the total number of PDs in the array. The total received power $P_{r_{tot}}$ by the array is given by Equation (18), where $P_{r_{\alpha_{i}}}$ is optical power received by the $i^{th}$ PD. Thus, the SNR may also be evaluated as Equation (19). For this study, the optical transmit power was set at $P_{T}=10$ W, and a BW=5 MHz has been used throughout. Here, we consider the modulation frequency to be fixed at the upper limit of the modulation bandwidth. This frequency is well within the cut-off bandwidth of the selected photodiode. The dynamic relationship of shot noise with received power is not emulated in this case, which should not affect the model’s accuracy because the contribution of shot noise where SNR ≈ 1 is infinitesimally small compared to those of thermal noise and dark current. The received power is estimated at the PD’s planar surface area resolved for the AOI, not considering its spectral sensitivity. These decisions were taken to better isolate and observe the geometrical implications of the array design from the influences of PD’s optical parameters, which are dependent on the manufacturer and choice of photodiode.

(17) $$P_{\eta_{tot}}=NEP\times \sqrt{BW}\times I_{PD}$$

(18) $$P_{r_{tot}}=\sum_{i=1}^{I_{PD}}P_{r_{\alpha_{i}}}$$

(19) $$SNR=\frac{P_{\eta_{tot}}}{P_{r_{tot}}}$$

The arrays were first analyzed under movement by horizontal and vertical displacements about the positive quadrant of an x-y cartesian plane, with the origin at the location of the LED emitter. One quadrant is deemed sufficient due to the symmetrical intensity profile about the center axis, collinear with the environment’s x-axis. The incremental displacement resolution in both directions was 0.1 m. The results were generated for both three-PD and five-PD arrays for rotations from −90° to +90° for all arrays about the array’s center coordinate in incremental intervals of 30°, for wavelengths of 450 nm (blue), 500 nm (green), and 550 nm (lime-green/yellow), to observe the received power while moving within the Jerlov seawater types. The corresponding attenuation parameters are in Table 3. Finally, a bit-error analysis was conducted for each of the previous wavelengths and Jerlov seawater types. Modulation schemes were selected that were popular for DLOS links such as NRZ-OOK, L-pulse position modulation (PPM, for L-values 4, 8, and 16), and binary-phase shift keying (BPSK). The results were compared against a forward-error correction (FEC) limit of $3.8\times 10^{-3}$. Their respective BERs were evaluated using Equations (20)–(22), where $erfc\left(\cdot \right)$ refers to the complimentary error function [30].

(20) $${BER}_{NRZ-OOK}=\frac{1}{2}erfc\left(\frac{\sqrt{SNR}}{2\sqrt{2}}\right)$$

(21) $${BER}_{L-PPM}=\frac{1}{2}erfc\left(\frac{1}{2\sqrt{2}}\sqrt{SNR\times \frac{L}{2}\times \log_{2}L}\right);\left\{L=4,\;8,\;16\right\}$$

(22) $${BER}_{BPSK}=\frac{1}{2}erfc\left(\sqrt{SNR}\right)$$

### 5.2. Algorithm

**Figure 5:**
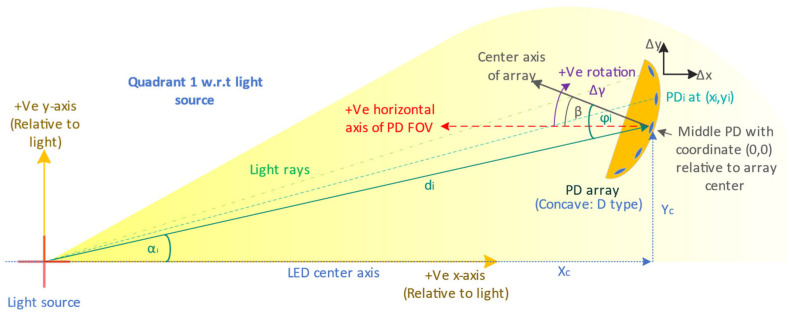
Each array is moved within the positive quadrant of a cartesian coordinate plane with the origin centered at the location of the light source. The results are generated for horizontal and vertical displacements.

The arrays are freely moving in the region along and above the center axis of the Tx, as shown in Figure 5. Algorithm 1 shows the pseudocode for determining the array received power, and the abbreviations of the parameters used are explained in the Abbreviations section.
**Algorithm 1** Array Received Power
**Input:** $c,\;\tau \left(\alpha \right)\;\left(0{}^{\circ}\leq \alpha \leq 90{}^{\circ}\right),\;n,\;A_{r},\;P_{T},\;I_{PD},\;\left(x_{0_{i}},y_{0_{i}}\right),\;\gamma_{0_{i}},x_{min},\;x_{max},\;\Delta x,\;y_{min},\;y_{max},\;\Delta y,\;$ Δγ
**Output:** $P_{r\left(x_{c},y_{c}\right)}$1:Calculate: $\left|r_{i}\right|=\sqrt{x_{0_{i}}^{2}+y_{0_{i}}^{2}\;}$
2:$\beta_{i}=\left(\gamma_{0_{i}}+\Delta \gamma \right)$3:$\omega_{i}=-\tan^{-1}\left(\frac{y_{0_{i}}}{x_{0_{i}}}\right)$4:Evaluate new relative coordinates of $i^{th}$ PD to middle PD: $\left(x_{\Delta \gamma_{i}},y_{\Delta \gamma_{i}}\right)=\left(-\left|r_{i}\right|\cos\left(\omega_{i}+\Delta \gamma \right),\left|r_{i}\right|\sin\left(\omega_{i}+\Delta \gamma \right)\right)$
5:$\left(x_{c},y_{c}\right)=\left(x_{min},y_{min}\right)$6:**while** $y_{c}<y_{max}$ **do**7: **while** $x_{c}<x_{max}$ **do**8:  **for** each i PD **do**9:   $d_{i}=\sqrt{{\left(x_{c}+x_{\Delta \gamma_{i}}\right)}^{2}+{\left(y_{c}+y_{\Delta \gamma_{i}}\right)}^{2}}$
10:   $\alpha_{i}=-\tan^{-1}\left(\frac{y_{c}+y_{\Delta \gamma_{i}}}{x_{c}+x_{\Delta \gamma_{i}}}\right)$
11:   $\varphi_{i}=\left(\alpha_{i}+\beta_{i}\right);\left(0{}^{\circ}\leq \varphi_{i}\leq 90{}^{\circ}\right)$
12:   $\left(x_{c},y_{c}\right)=\left(x_{c}+\Delta x,y_{c}+\Delta y\right)$
13:   $I_{0}=\left(\frac{P_{T}}{2\pi d_{i}^{2}\sum_{n=1}^{N}\tau_{\alpha_{n}\left(\cos\alpha_{n-1}-\cos\alpha_{n}\right)}}\right)$
14:   $P_{r_{\alpha_{i}}}=\tau_{\alpha_{i}}I_{0}e^{-cd_{i}}A_{r}\cos\left(\varphi_{i}\right)$
15:   Total power: $P_{r\left(x_{c},y_{c}\right)}=\sum_{i=1}^{I_{PD}}P_{r_{\alpha_{i}}}$
16:  **end for**17:  $\left(x_{c},y_{c}\right)=\left(x_{c}+\Delta x,y_{c}\right)$
18: **end while**19: $\left(x_{c},y_{c}\right)=\left(x_{c},y_{c}+\Delta y\right)$
20:**end while**

## 6. Results and Discussion

### 6.1. Organization of Results

**Figure 6:**
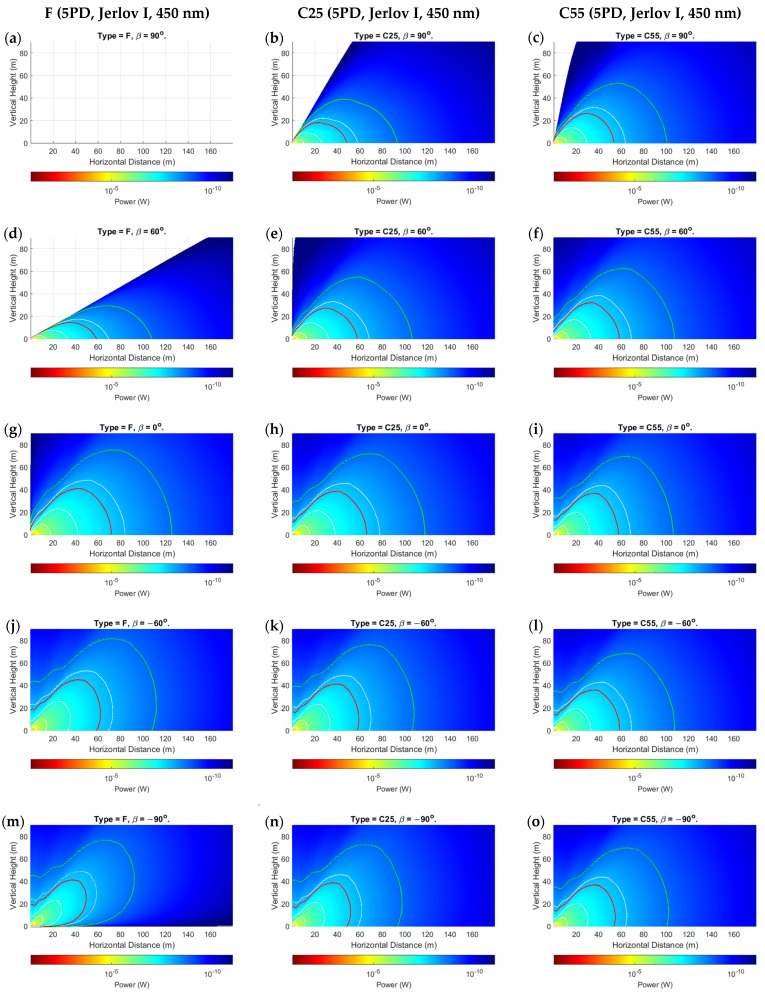
$P_{r_{tot}}$ for 5PD arrays at: (**a**–**c**) *β* = 90°; (**d**–**f**) *β* = 60°; (**g**–**i**) *β* = 0°; (**j**–**l**) *β* = −60°; (**m**–**o**) *β* = −90° for Jerlov I; c=0.022 m^−1^. Red and green lines are 10- and 0-dB contours, respectively.

**Figure 7:**
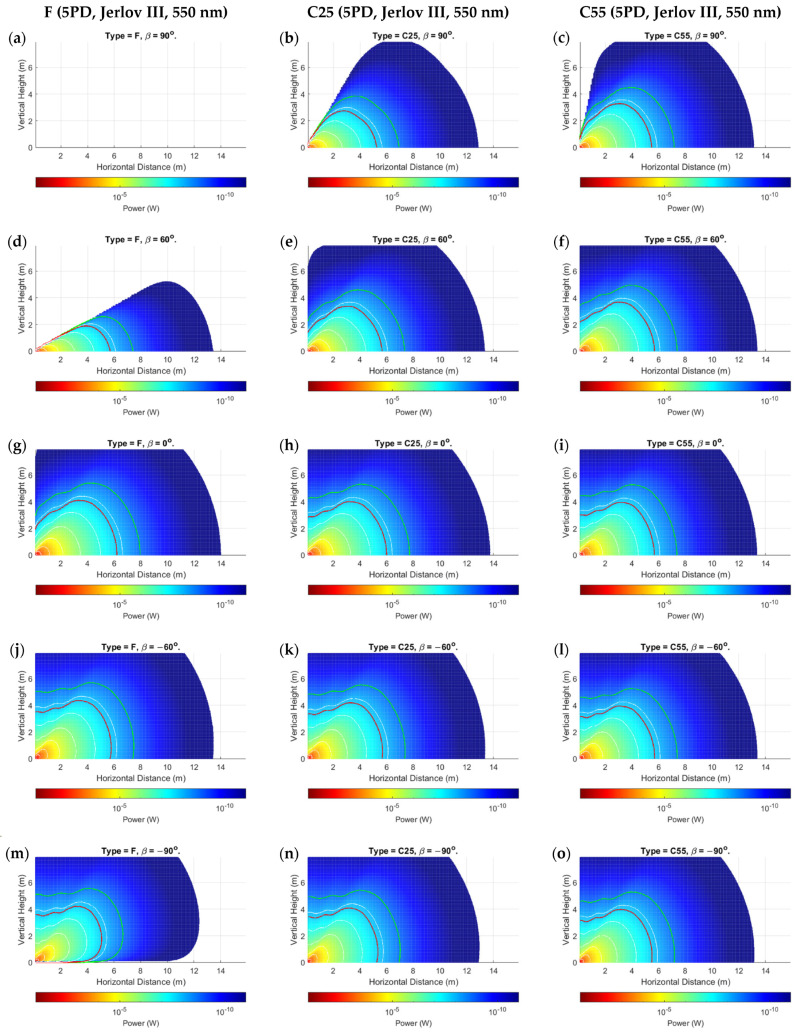
$P_{r_{tot}}$ for 5PD arrays at: (**a**–**c**) *β* = 90°; (**d**–**f**) *β* = 60°; (**g**–**i**) *β* = 0°; (**j**–**l**) *β* = −60°; (**m**–**o**) *β* = −90° for Jerlov III; c=1.045 m^−1^. Red and green lines show 10- and 0-dB contours, respectively.

**Figure 8:**
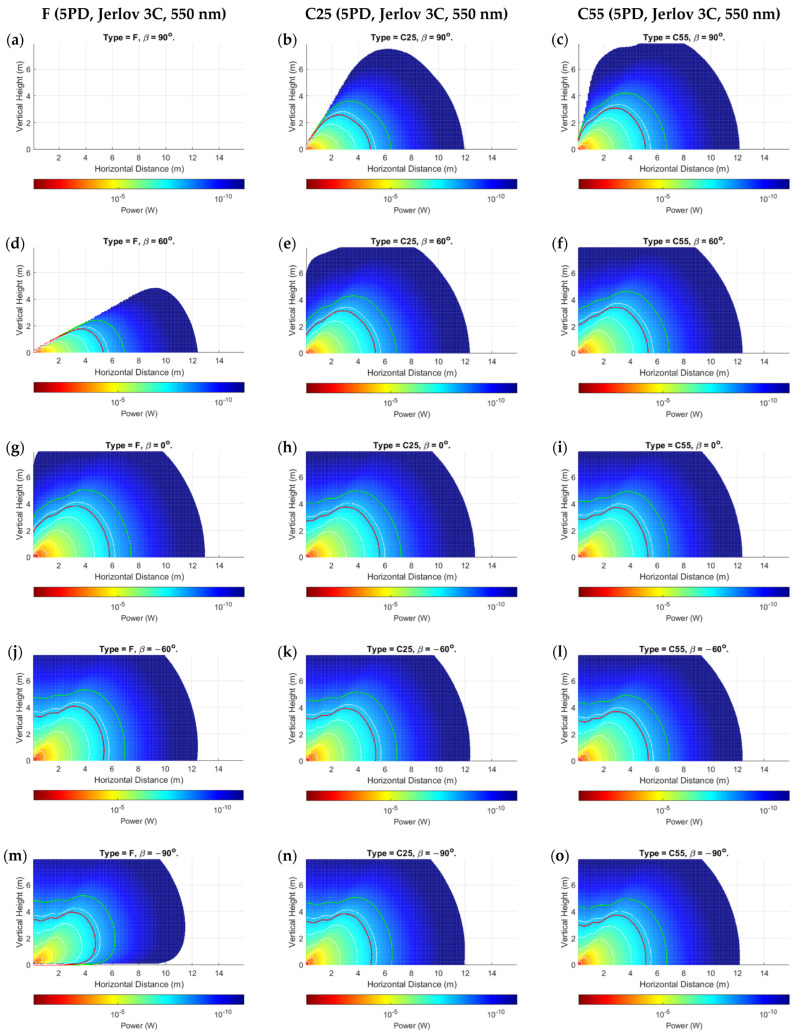
$P_{r_{tot}}$ for 5PD arrays at: (**a**–**c**) *β* = 90°; (**d**–**f**) *β* = 60°; (**g**–**i**) *β* = 0°; (**j**–**l**) *β* = −60°; (**m**–**o**) *β* = −90° for Jerlov 3C; c=1.142 m^−1^. Red and green lines show 10- and 0-dB contours, respectively.

**Figure 9:**
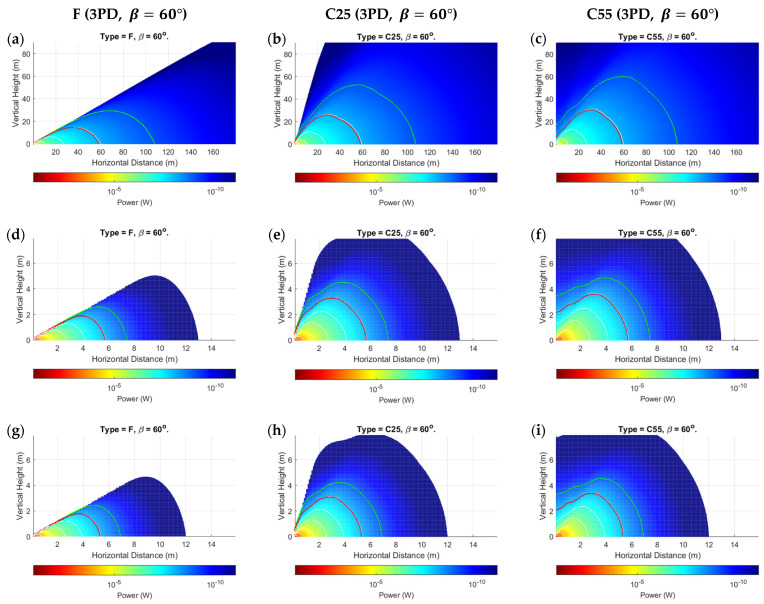
$P_{r_{tot}}$ for respective 3PD arrays at *β* = 60° rotation in: (**a**–**c**) Jerlov I (*λ* = 450 nm); (**d**–**f**) Jerlov III (*λ* = 550 nm) and (**g**–**i**) Jerlov 3C (*λ* = 550 nm). Red and green lines show 10- and 0-dB contours, respectively.

The proposed power distribution model demonstrated results that validated its applicability, while also highlighting some nuanced observations for mobile UOWCs that should not be ignored. We evaluated all scenarios as outlined in Section 5.1, but only showed the more significant results due to space constraints. Figure 6, Figure 7 and Figure 8 present the spatial distribution of received power for 5PD arrays in the x-y cartesian plane for the three configurations, F, C25, and C55, for rotations of β=−90° to β=90° in 30° increments. Figure 6 shows the results for Jerlov type I for a transmission wavelength of λ=450 nm, and Figure 7 and Figure 8 for Jerlov types III and 3C, respectively, for λ=550 nm. Figure 9 provides results for the 3PD arrays, F, C25, and C55, for Jerlov types I, III, and 3C at β=60°. This rotation angle was specifically chosen to display the trend in the blind spots across the array designs, discussed in Section 6.2.

Jerlov I and III were provided as they are the two extremes of his oceanic classifications, and Jerlov 3C is provided as the extreme for coastal classification. Results are not provided beyond Jerlov 3C due to very high attenuation coefficients that signify UOWCs may be impractical in these environments. Results are displayed for λ=450 nm for Jerlov I and λ=550 nm for Jerlov III and 3C in Figure 6, Figure 7 and Figure 8 due to experiencing the least attenuation in respective water conditions as per Table 3.

**Figure 10:**
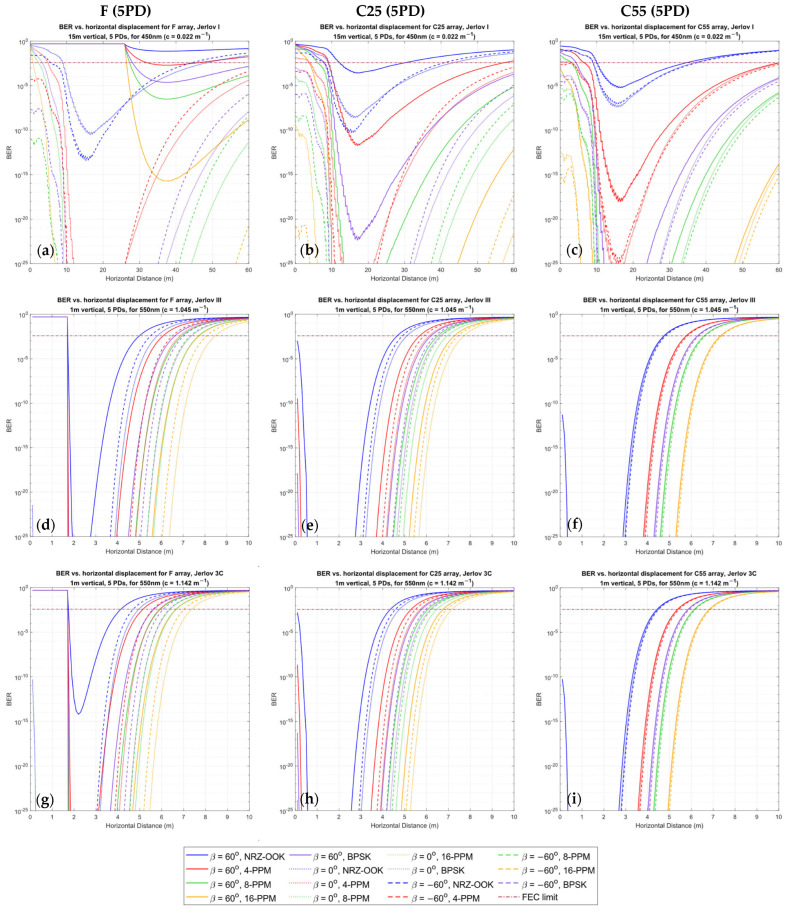
BER analysis for respective 5PD arrays in: (**a**–**c**) Jerlov I; (**d**–**f**) Jerlov III; and (**g**–**i**) Jerlov 3C. A wavelength of 450 nm was used for Jerlov I and 550 nm was used for Jerlov III and 3C, respectively, due to them being the least attenuated light wavelengths in the respective water types, as per Table 3. Jerlov I was modeled for a 15 m vertical displacement, and Jerlov III and 3C at a 1 m vertical displacement.

In Figure 6, Figure 7 and Figure 8, the green line shows the spatial points where SNR≈0 dB, and the red line corresponds to SNR≈10 dB. This information is helpful to draw comparisons between them and the BER results provided for the 5PD arrays in Figure 10. In communication theory, it is known that a minimum SNR of 10 dB is required for an NRZ-OOK scheme; hence the red line can be approximated as the region where the NRZ-OOK scheme reaches the FEC limit. Each consecutive white line shows the spatial points of equal received power where the received power decreases beyond a $10^{-\varsigma }$ W limit, where ς is the line number counted from the point of origin, moving away from the emitter.

**Figure 11:**
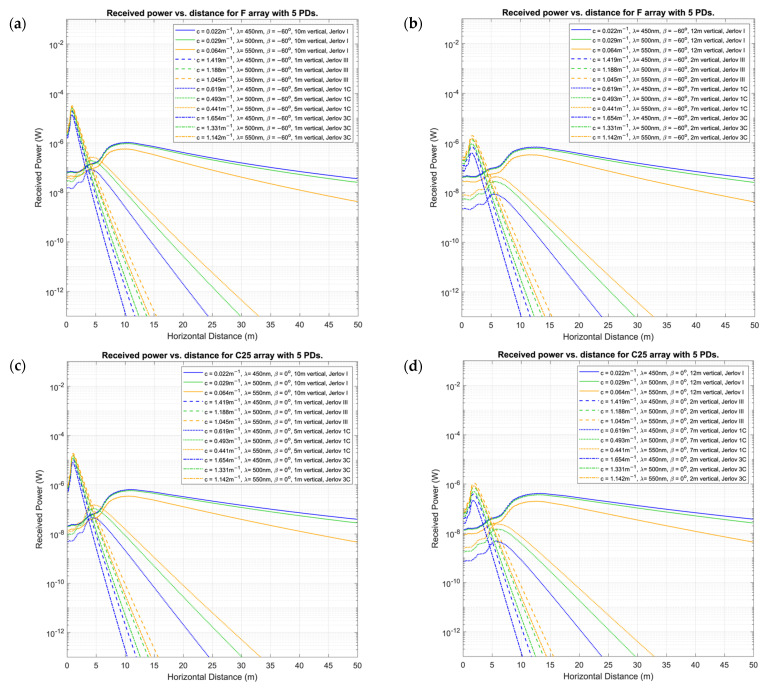

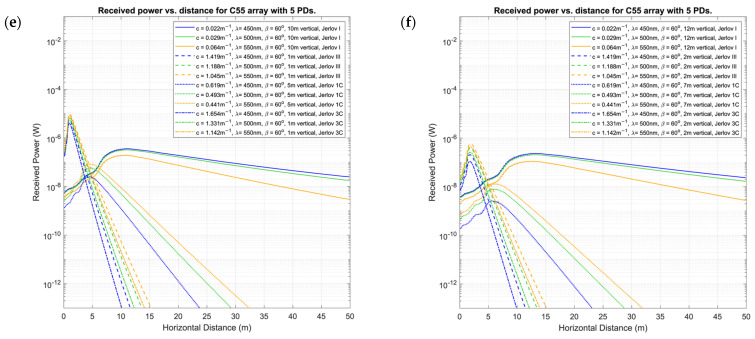
$P_{r_{tot}}$ for 5PD arrays of: (**a**,**b**) type F; (**c**,**d**) type C25; and (**e**,**f**) type C55; at β={−60°, 0°, 60°} respectively, for Jerlov I, III, 1C, and 3C, for λ={450, 500, 550} nm.

Finally, Figure 11 provides 2D snapshots of received power for a pair of select side-by-side vertical displacements for $\lambda =\left\{450,\;500,\;550\right\}$ nm under rotations of $\beta =\left\{60{}^{\circ},\;0{}^{\circ},-60{}^{\circ}\right\}$ for Jerlov types I, III, 1C, and 3C. Results for λ=500 nm and Jerlov 1C are included in addition to the previous results to support suggestions made in Section 6.2. In the subsequent analysis, we refer to α as the overall angle of elevation between the PD array and LED.

### 6.2. Discussion

As a directly visible observation, the F-type arrays of both three and five PDs show a complete blind region whenever (α+β)≥90°, which can be seen in the subfigures (a) and (d) of Figure 6, Figure 7 and Figure 8 and in Figure 9a,d,g. Comparisons between the corresponding 5PD, C55, C25, and F arrays showed that this blind region is more significant for flatter arrays. At β=90°, the C55 array performed the best, whereas at $\beta =-90^{{}^{\circ}}$, the C25 and C55 arrays seemed to compete for performance based on their location. This is expected as the channel model given by Equation (16) dictates that the AOI should be less than 90°, where the more curved C55 array benefits from a greater spread of FOVs. This is a disadvantage for flat arrays as it shows that complete link loss could result if the Rx was suddenly disoriented due to UUV collision with fish or objects in the underwater environment. Having all PDs oriented in the same direction may also cause a drastic, unreliable BER response as shown in Figure 10a. Nonetheless, the F-type array showed the maximum received power when β≤0°, in the regions at which α≈ β when compared to the other array types at the same β and in the same water condition. This is due to the AOI reaching 0° to planar PD surfaces in the F-type array. However, it is observed that the locations where maximum power is attained by the F-type array are not along the α=β angle but along a direction where the combined effects of the resolved AOI component and LED intensity distribution have achieved an optimal level. This is visible in subfigures (j) and (m) of Figure 6, Figure 7 and Figure 8. Although this may also be true for the C25 and C55 configurations, it cannot be determined so easily due to greater individual PD FOV spreads. However, when β=0°, the α=0° direction for the F-type array produced the greatest overall power across all water types and in both 3PD and 5PD configurations due to also observing the greatest light intensity along the horizontal axis. This variability factor may be better addressed from the Tx side by designing a Tx that emits uniform intensity distribution. However, more spread-out configurations fared much better for β>0° across all arrays than their flatter counterparts, regardless of the number of PDs in the array, as seen when comparing 3PD C55 results in Figure 9c,f,i with their corresponding 5PD F and C25 results in Figure 6, Figure 7 and Figure 8.

Analysis of the individual received powers by the PDs in each array configuration showed that the separation distance between PDs played a lesser role as the distance from Tx increased. There is no noticeable difference between C- and D-type arrays of equal vertex depth. This may be due to their equivalent overall FOVs. However, convex arrays are recommended overall due to the minimized effect of shadowing by the solid housing. The effect of having more PDs in the array becomes less significant at greater distances, possibly owing to the much greater geometric loss compared to the collective photodetector surface area. It is more important for PDs to maintain their orthogonality to the incoming ray at any location. This is observed clearly by comparing each 3PD subgraph from Figure 9 with their corresponding 5PD counterparts from Figure 6, Figure 7 and Figure 8, where there was minimal improvement even at short range (referring to turbid profiles) where the geometric spread is less. This shows that the behavior of a fixed-geometry array tends to resemble a point receiver, with FOV equivalent to the sum of FOVs of the individual PDs as the range from Tx increases. This simplifies the power distribution analysis in a 3D space for an array populated along 3D axes, as applying rotations to only the roll, pitch, and yaw on the planes of the PDs (ignoring their displacement vectors) about the array’s center coordinate would yield a near-accurate response, while the intensity distribution remains governed by the parameter α.

Ripples of received power were observed in the regions beyond the viewing angle of the LED (α≥50°), where the combined effects of the PDs emerging into the light field and the irregularities in intensity distribution produce waves of high and low power along the horizontal distance at a given height, until it reaches a peak, before declining thereafter. This is visible in Figure 11. The horizontal distance at which this peak occurs increases with increasing vertical height from Tx. Figure 6, Figure 7 and Figure 8 further show a considerable connectivity distance is possible in this region, which is beyond the beam-spread angle θ, although the intensity is less than 50% of $I_{0}$ in this region and the AOI to the PDs is small.

One of the more interesting observations is that in clearer waters (Figure 6 and Figure 9a–c), for any given α, the consecutive separations between the white lines increase. In the turbid profiles (Figure 7, Figure 8 and Figure 9d–i), this separation is more consistent, except when α is almost 90° or the location is very close to the Tx. This could be due to the very low influence of attenuation in Jerlov I compared to the effect of geometric loss. As seen in Figure 6, the separation between the Tx and 0 dB boundary spans almost twice the distance as the distance from the Tx to the 10 dB line. This signifies that for clear water UOWCs, although NRZ-OOK may be effective, a more bandwidth-efficient scheme such as higher-order PPM schemes may offer longer connectivity. This is corroborated by the BER results in Figure 10. As turbidity increases, the boundary for NRZ-OOK reaches much closer to the 0 dB line, and therefore using more complex modulation schemes may not be so beneficial.

Considering BER, Figure 10 shows that with greater vertical distance, as the horizontal distance increases, the BER curve drops from a high value to a minimum, before increasing again to pass the FEC threshold. This is due to the lower light intensity experienced at these locations as well as some PDs of the arrays that have yet to come into view of the light rays. In the F-array, this complete blind spot causes the “flat” lines in Figure 10a,d,g for β=60°. The minima are where all contributing factors have reached an optimal level. In Figure 10a, the locations of the minima in the F-array are distributed across the space. C55 (Figure 10c), on the other hand, shows shallower minima across all β, but their locations are more predictable and the BER response was also more consistent.

The F-type arrays produce two loci with a significant distance in between, where the received power is at the same level due to a form of cresting about the viewing angle of the LED, which could be due to the combined effect of the variation of relative intensity distribution and the PD’s angular sensitivity distribution both reaching optimums for output power. If 3D intensity distribution is visualized, two circular loci of the same power level would have been seen around the center axis of the Tx. It might be preferable to eliminate this effect entirely by using two wavelengths of light that experience two different spectrum-dependent attenuations, whose difference in received power increases consistently with distance. This is observed in Figure 11, where the received power is modeled at the Rx for clear ocean Jerlov types I (10 m and 12 m vertical displacements) and III (1 m and 2 m vertical displacements), and coastal types 1C (5 m and 7 m vertical displacements) and 3C (1 m and 2 m vertical displacements). Here, it is observed that for all four water types, the difference in received power between each wavelength is widening consistently by a logarithmic factor with horizontal distance, irrespective of orientation. If the rate of change of this difference could be computed in real time, it could enable UUVs to determine water-condition-related attenuation while traversing through inhomogeneous environments such as in vertical channels of the euphotic zone. Using two spectral wavelengths could also help to isolate the distance attenuation factor $e^{-c\left(\lambda \right)d}$ in Equation (16) from the orientation effect of the PD array. This feature may be advantageous for employing optical received-signal strength-indicator-based localization.

Coastal types beyond Jerlov 3C showed an extremely rapid decline of the received power at very short range, implying that UOWCs in these environments may not be beneficial. However, this model cannot assess the effects of the light multipaths in these highly scattered turbid environments. For this, a Monte Carlo simulation may be more appropriate. This is due to a Monte Carlo simulation’s ability to analyze outcomes from the level of a single photon. This could account for the geometric implications of array geometry towards the distribution of delay spreads in highly scattering environments. We further acknowledge that the simulation results provided here need to be verified through experimentation. It is possible to recreate the Jerlov types artificially through scattering and absorption agents, such as by using Maalox antacid [31,32]. The 3PD and 5PD arrays can be designed and developed in the described F, C25, and C55 configurations. However, attaining an equal level of geometric loss requires an area as analyzed in the simulations, which is only possible in the real ocean. Instead, selecting LEDs of 450 nm, 500 nm, and 550 nm that provide a similar intensity distribution, but emit a scaled-down fraction of optical power may be ideal. Using micro-LEDs may be a good option. We propose such a test as future work.

In summary, we highlight these recommendations for PD array designs:

- Flat array designs are optimal for links where Tx–Rx alignment can be strictly maintained. This will need to be controlled dynamically as the UOWC moves to maintain orthogonality to the light ray. If not, the response may be very unpredictable and disadvantageous. There is also a significant risk of total blindness.
- Curved arrays are good overall for mobile UOWCs as they have large overall FOVs. This produces more predictable outcomes and, overall, less drastic BER variation. However, the spread-out PD configurations mean that there will always be some PDs at non-optimal AOIs to light. This may cause an irregular high-and-low received-power response until a peak power is observed at a certain horizontal distance for a given height from the Tx. The horizontal location of this peak power increases with increasing distance.
- The number of PDs used in the array plays a less significant role in optimal performance than the overall FOV of the array, especially as the distance from the transmitter increases. Overall, a convex shape may be better for curved arrays.
- As the clarity of the water improves, the range of link coverage could be improved much more significantly by utilizing more bandwidth-efficient modulation schemes.
- In the presence of non-uniform optical intensity distributions, the optimal BER location for all flat and curved arrays in mobile UOWCs will be sandwiched between two or more high-BER regions contingent on the intensity profile and angular distribution of the PDs.
- Where designers may want to address localization via the optical link, using received-signal-intensity (RSSI)-based methods may not be feasible as multiple loci produce equal received power. However, isolating the attenuation factor by using two or more distinct wavelengths may be a beneficial strategy.

Considering the above, we emphasize the need for array designs that can shape-shift dynamically between flat and convex geometry for the received power in a mobile UOWC to remain optimized. We recognize that this may present challenges in designing housing that can move as well as withstand deep-sea pressures. It will require future research attention.

## 7. Conclusions

In this study, we developed and presented an analytical model to better represent the non-uniform intensity distribution of diffused light emitters for mobile UOWC systems. Thereafter, using the model, we investigated the effects of PD placement and orientation towards achieving optimal UOWC links using arrays of varying levels of concavity or convexity. Our findings suggest that flat array designs are best when strict alignment is maintained continually. However, this may require dynamic adjustment to prevent otherwise unpredictable responses and even complete signal loss. Curved arrays offer larger FOV, leading to more predictable performance, but the spread-out PD configurations mean that there will be some PDs at non-optimal AOI at any given time. This causes irregular received power responses closer to the transmitter. Furthermore, as the clarity of the water improves, the range of link coverage could be improved much more significantly by utilizing more bandwidth-efficient modulation schemes. Where designers may want to integrate localization with the UOWC, using two or more transmission spectra may be more helpful for RSSI-based localization. We also show that it is possible to achieve connectivity beyond the beam-spread angle of the LEDs. As an overall recommendation for designing receivers, we highlight the need for dynamically shape-shifting PD arrays. Future work will further explore the validation of the presented results by means of controlled experiments in a tank-based setting.

## Figures and Tables

**Table 2:** Nominal values of absorption (a), scattering (b), and attenuation (c) coefficients for different water types [3].

Water Type	*a* (m^−1^)	*b* (m^−1^)	*c* (m^−1^)
Clear ocean	0.114	0.037	0.151
Coastal ocean	0.179	0.220	0.339
Turbid harbor	0.366	1.829	2.195

**Table 3:** Spectral attenuation coefficients of the different Jerlov water types for 450 nm, 500 nm, and 550 nm wavelengths [25].

Jerlov Type	$c_{\lambda =450nm}$ (m^−1^)	$c_{\lambda =500nm}$ (m^−1^)	$c_{\lambda =550nm}$ (m^−1^)
Jerlov I	0.022	0.029	0.064
Jerlov IA	0.028	0.033	0.066
Jerlov IB	0.092	0.088	0.116
Jerlov II	0.528	0.450	0.420
Jerlov III	1.419	1.188	1.045
Jerlov 1C	0.619	0.493	0.441
Jerlov 3C	1.654	1.331	1.142
Jerlov 5C	2.167	1.711	1.447
Jerlov 7C	3.842	3.050	2.545
Jerlov 9C	5.333	4.213	3.468

## Data Availability

Data are contained within the article.

## Abbreviations

c	Attenuation coefficient
$\tau \left(\alpha \right)$	Relative intensity of light at an angle α
n	Number of annuli
$A_{r}$	Planar surface area of a PD
$P_{T}$	Output optical power of emitter
i	Number of PDs in array
$I_{PD}$	Total number of PDs in array
$\left(x_{0_{i}},y_{0_{i}}\right)$	i^th^ PD’s coordinate relative to middle PD $\left(x_{c},y_{c}\right)$
$\gamma_{0_{i}}$	i^th^ PD’s inclination relative to middle PD axis
$x_{min},x_{max}$	Minimum and maximum horizontal test limit
$y_{min},y_{max}$	Minimum and maximum vertical test limit
Δx	Horizontal distance increment interval
Δy	Vertical height increment interval
Δγ	Rotation angle of array
